# A novel *ERAP2* haplotype structure in a Chilean population: implications for ERAP2 protein expression and preeclampsia risk

**DOI:** 10.1002/mgg3.13

**Published:** 2013-05-30

**Authors:** Derek L Vanhille, Lori D Hill, DaShaunda D Hilliard, Eun D Lee, Maria E Teves, Sindhu Srinivas, Juan P Kusanovic, Ricardo Gomez, Efstratios Stratikos, Michal A Elovitz, Roberto Romero, Jerome F Strauss

**Affiliations:** 1 Department of Obstetrics and Gynecology and the Center on Health Disparities, Virginia Commonwealth University School of MedicineRichmond, Virginia, 23298; 2 Maternal and Child Health Research Program, Department of Obstetrics and Gynecology, University of Pennsylvania School of MedicinePhiladelphia, Pennsylvania, 19104; 3 Perinatology Research Branch, NICHD/NIH/DHHSDetroit, Michigan and Bethesda, Maryland; 4 Department of Obstetrics and Gynecology, Sótero del Río HospitalSantiago, Chile; 5 Department of Obstetrics and Gynecology, Pontificia Universidad Católica de ChileSantiago, Chile; 6 National Centre for Scientific Research “Demokritos” INRASTES, Agia Paraskevi Attikis15310, Athens, Greece

**Keywords:** African-Americans, Chileans, ERAP2, haplotype, preeclampsia

## Abstract

Single nucleotide polymorphisms (SNPs) in the endoplasmic reticulum aminopeptidase 2 (*ERAP2*) gene are associated with preeclampsia (PE) in different populations. rs2549782, a coding variant (N392K) that significantly affects substrate specificity, is in linkage disequilibrium (LD) with rs2248374, a marker SNP associated with ERAP2 protein expression in previously studied populations. As a result of nonsense-mediated RNA decay, ERAP2 protein is not expressed from the rs2248374 G allele. We previously reported that the fetal rs2549782 minor G allele is associated with PE in African-Americans, but not in Chileans. In this study, we found that rs2549782 was in LD with rs2248374 in African-Americans, but not in Chileans. The unexpected lack of strong LD in Chileans raised the possibility that rs2248374 could be associated with PE in the absence of an association with rs2549782. However, we found no significant association for this allele with PE in Chileans. Chileans homozygous for the rs2248374 G allele did not express 110 kDa ERAP2 protein, consistent with nonsense-mediated RNA decay, and carriers of the rs2248374 A allele did. We conclude that the Chilean *ERAP2* haplotype structure allows for the expression of the major T allele of rs2549782 encoding 392N, which could impact peptide trimming and antigen presentation. Our discovery of racial differences in genetic structure and association with PE reveal heretofore unrecognized complexity of the *ERAP2* locus.

## Introduction

Preeclampsia (PE), a complex disorder specific to pregnancy, is a major cause of maternal and perinatal morbidity and mortality worldwide. From 3% to 8% of pregnancies are affected by PE, and there is strong evidence pointing to genetic risk factors (Anoymous 2002). Candidate genes studied to date have different potential pathophysiological roles related to endothelial function, inflammation, vascular tone, immunogenetics, hemostasis, metabolism, and oxidative stress (Williams and Broughton Pipkin 2011). Endoplasmic reticulum aminopeptidase 2 (ERAP2), a 110 kDa glycoprotein, is thought to be involved in immune responses, serving as a trimming enzyme involved in antigen presentation. The enzyme has also been implicated in inflammation and blood pressure control through proteolysis of vasoactive proteins (Tsujimoto and Hattori 2005; Evnouchidou et al. 2012). ERAP2 is expressed in the placental syncytiotrophoblast, making it an appealing PE candidate gene (Fruci et al. 2008; Zhang et al. 2010).

Certain *ERAP2* single nucleotide polymorphisms (SNPs) have been reported to be associated with an increased risk for PE in different ethnic populations, including rs2549782 (Founds et al. 2009; Johnson et al. 2009; Hill et al. 2011a). This SNP has also been reported to be in linkage disequilibrium (LD) with rs2248374, rs2548538, rs2287988, and rs1056893; all marker SNPs that characterize two haplotypes (A & B) which are associated with ERAP2 protein expression in populations that have been previously studied (Andrés et al. 2010). The *ERAP2* haplotype B is associated with a splice-site variant caused by the rs2248374 SNP major allele that is thought to result in nonsense-mediated RNA decay, which precludes protein expression (Tanioka et al. 2003; Coulombe-Huntington et al. 2009). Indeed, the 110 kDa ERAP2 protein is not detectable in tissues from individuals homozygous for the rs2248374 G major allele. Individuals homozygous for the minor A allele express twice as much ERAP2 protein as those heterozygous for this SNP (Andrés et al. 2010).

The minor allele of rs2549782 causes a nonconservative amino acid substitution (N392K), which has been shown to alter ERAP2 enzyme activity and substrate specificity (Evnouchidou et al. 2012). However, because of the LD with rs2248374 and the haplotype structure of *ERAP2*, rs2549782 expression is monoallelic (Bjornsson et al. 2008; Song et al. 2012; Lee et al. 2013) and only the G minor allele, which encodes the ERAP2 392K amino acid variant, is expressed. Thus, in populations in which the rs2549782 and rs2248374 SNPs are in LD, the “wild-type” protein encoded by the rs2549782 T allele is never produced.

We have previously examined genotypes for rs2549782 in African-American and Chilean women and their offspring in a case–control study to test for associations with PE (Hill et al. 2011a). We discovered a significant association of the fetal (neonatal) rs2549782 G minor allele and PE in African-Americans, but not in Chileans (Hill et al. 2011a). The present study was conducted to evaluate the haplotype structure in the populations that we studied to determine if the divergent findings in African-Americans and Chileans could be explained by different genetic structures. In this study, we discovered that the Chilean population has a unique *ERAP2* haplotype structure in which rs2549782 is not in strong LD with the other four SNPs of the haplotype, including rs2248374, as predicted from the analysis of all other populations previously studied (Andrés et al. 2010). In contrast, the African-American population did demonstrate the anticipated LD between these two SNPs. The lack of LD observed in the Chilean populations allows for the expression of the major T allele of rs2549782 because of its ability to be paired with the minor A allele of rs2248374. However, our study failed to detect the homozygote TT genotype of rs2549782 in combination with the homozygote AA genotype of rs2248374, raising the possibility that the homozygous state for the allele encoding 392N of ERAP2 may not be tolerated.

## Materials and Methods

### Subjects

The Chilean population (maternal and neonatal dyads) studied in the present work has been described previously (Hill et al. 2011a). It consisted of cases (women with PE) and their neonates (*n* = 528 dyads); and controls (women who delivered at term with a normal pregnancy outcome) and their neonates (*n* = 575 dyads). The Chilean population is estimated at nearly 95% white and mestizo (mixed white and Amerindian); 3% Amerindian; and 2% other. Mixtures between the conquering Spaniards, largely Andalusians and Basques, and the Mapuches (Araucanians) produced the principle Chilean racial type (2002 census).

The African-American population has also been described, with the exception that additional cases and controls were added yielding 382 maternal cases and 342 maternal controls and 511 fetal (neonatal) cases and 702 neonatal controls through recruitment at the Hospital of the University of Pennsylvania in Philadelphia and the Hutzel Women's Hospital in Detroit. Of the 1937 total African-American samples, 68% were paired maternal–fetal dyads.

PE was defined based on the presence of gestational hypertension (systolic blood pressure ≥140 mmHg and/or diastolic blood pressure ≥90 mmHg) and proteinuria (≥300 mg in a 24-h urine collection, two or more dipstick measurement of 1+, or one or more dipstick measurement ≥2+) according to ACOG (Anoymous 2002) and the National High Blood Pressure Education Program (Anoymous 2000). Patients were considered to have a normal pregnancy outcome if they did not have any medical, obstetrical, or surgical complication, and delivered a term neonate (≥37 weeks) of appropriate birth weight for gestational age without complications.

### Sample collection

Maternal blood samples were obtained from the mother at the time of enrollment in the protocol. Umbilical cord blood samples or neonate cheek swabs were obtained immediately after delivery. Blood samples were collected with a vacutainer into tubes containing ethylenediaminetetraacetic acid. The plasma tubes were balanced and centrifuged at 1300*g* for 10 min at 4°C to separate cellular components from clear plasma, and the samples were stored at −70°C until assay.

### DNA extraction

DNA was extracted from maternal and cord blood with the Qiagen Autopure system using standard procedures (Qiagen). DNA was extracted from neonate cheek swabs using traditional methods as previously described (Wang et al. 2004). For the 15 Chilean placental samples used for western blot analysis, DNA was extracted from the corresponding cord blood with the QIAquick PCR Purification Kit (Qiagen, Germantown, MD) using standard protocols.

### Genotyping

All PCR reactions contained 5–45 ng of DNA, 6.25 μL TaqMan Universal Master Mix (Applied Biosystems, Branchburg, NJ) (2×), 0.31 μL TaqMan Genotyping Assay (Applied Biosystems, Foster City, CA) (40×), and water for a final volume of 12.5 μL. Real-time allelic discrimination was performed using an ABI 7500 Fast Real-Time PCR Machine (Applied Biosystems, Foster City, CA) with the following settings: 50°C for 2 min, 95°C for 10 min, and 40 cycles of amplification (95°C for 15 sec and 60°C for 1 min). SNP genotyping and analysis were performed using the following five *ERAP2* TaqMan SNP Genotyping assays: C_3282749_20 for SNP rs2549782, C_25649530_10 for SNP rs2548538, C_25649529_10 for SNP rs2248374, C_25649516_10 for SNP rs2287988, and C_3282728_20 for SNP rs1056893. Allelic discrimination software determined each genotype via the use of VIC- or FAM-labeled probes (Applied Biosystems, Foster City, CA) as previously described (Hill et al. 2011a).

### Western blotting

Proteins were extracted from placental basal plate tissues using 500 μL of radioimmunoprecipitation assay buffer (150 mM NaCl, 1%15 Triton X-100, 0.5% sodium deoxycholate, 0.1% SDS, 50 mM Tris, pH 8.0). Equal amounts of protein (50 μg/lane) were heated to 95°C for 10 min in sample buffer, loaded onto 7.5% (for ERAP2) or 6.0% (for ERAP1) SDS-PAGE gels, separated by electrophoresis, and transferred to PVDF membranes (Millipore, Billerica, MA) by semidry transference. A dual-color precision plus protein standards (BIO-RAD, Hercules, CA) was used. Membranes were blocked for 1 h in 5% milk-TTBS (BIO-RAD, Hercules, CA) and then incubated overnight with goat anti-human Aminopeptidase LRAP/ERAP2 antibody (R&D Systems, Minneapolis, MN; 1:2000), mouse anti-human/mouse Aminopeptidase PILS/ARTS1 antibody (R&D Systems, Minneapolis, MN; 1:2000), or rabbit anti-β-actin antibody (Loading control, Cell Signaling Technology; 1:2000). After several washes in TTBS, the membranes were incubated with anti-goat IgG, anti-mouse IgG, or anti-rabbit IgG horseradish-peroxidase labeled antibodies (1:2000 dilutions) for 1 h at room temperature. Protein was detected with Super Signal Chemiluminescent Substrate (Pierce, Rockford, IL).

### Genetic structure analysis

Inter-SNP LD and haplotype block calculations were performed in Haploview using the default parameters for confidence intervals, the four gamete rule and solid spine analysis (Barrett et al. 2005) (version 4.2). Haplotype structure for the African-American and Chilean populations was determined by genotyping samples for the following five SNPs: rs2549782, rs2548538, rs2248374, rs2287988, and rs1056893. Analysis of 100 paired maternal–fetal samples in the African-American population (*n* = 200) did not show a haplotype structure differing from what had been previously reported (Andrés et al. 2010) so no additional samples were tested. Initial analysis of a subset of 100 paired maternal–fetal samples in the Chilean population (*n* = 200) showed larger variation in linkage between SNPs compared to previously reported populations (Andrés et al. 2010) so the full set of Chilean fetal samples (*n* = 1100) was analyzed to clarify the genetic structure. Fetal samples were chosen based on the previously reported fetal SNP association with PE in the African-American population (Hill et al. 2011a).

### Statistical analysis

Chi-squared tests implemented in R were used to test for differences in rs2248374, rs2549782 compound genotype counts between Chilean fetal cases and controls. Chi-squared tests implemented in R were used to test for differences in the number of observed versus expected compound genotypes of rs2248374 and rs2549782 in Chilean fetal, maternal, and total samples. Expected genotype counts were based on the observed allele frequencies. For example, the observed frequency of the rs2248374 A allele in Chilean fetal (neonatal) samples was 0.34 and the observed frequency of the rs2548792 T allele was 0.67, resulting in an expected AA,TT frequency of 0.34 × 0.34 × 0.67 × 0.67 = 0.052. A power calculation implemented in R was used to determine our power to detect the AA,TT (rs2248374, rs2549782) compound homozygote in the total Chilean population (fetal + maternal). Fisher's exact tests implemented in Haploview (Barrett et al. 2005) (version 4.2) were used to test individual SNPs and haplotypes for allelic associations with case–control status and to confirm Hardy–Weinberg equilibrium (HWE). These results were confirmed in the PLINK software package (Purcell et al. 2007). Chi-squared tests implemented in R were used to test for differences between genotype counts in cases and controls. A Bonferroni corrected *P*-value was used to account for multiple testing errors. Multiple logistic regression in R and epistasis testing methods implemented in PLINK (Purcell et al. 2007) were used to determine whether an interaction between rs2549782 and rs2248374 was associated with PE in Chilean fetal samples.

## Results

### *ERAP2* SNP allele frequencies in African-American and Chilean populations

**Figure 1:**
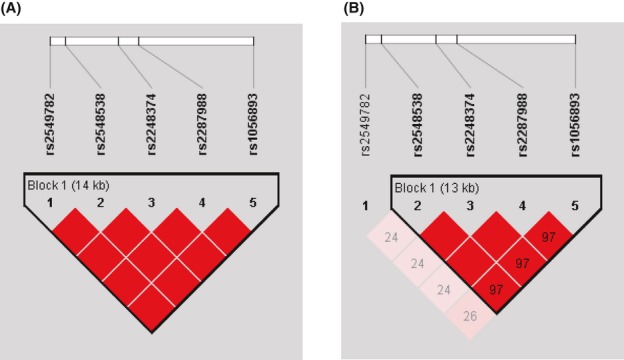
Linkage disequilibrium (LD) plots for *ERAP2* in African-American and Chilean fetal (neonatal) populations. (A) Plot for five SNPs in LD in the African-American population (SNP order: rs2549782, rs2548538, rs2248374, rs2287988, rs1056893). (B) Plot for four SNPs in LD in the Chilean population (SNP order: rs2548538, rs2248374, rs2287988, rs1056893). *R*-squared values are displayed within blocks. Dark red blocks without a number displayed represent *R*-squared = 1.00 and *D*' = 1.00. *D*' values for pink blocks ranged from 0.52 to 0.54. ERAP2, endoplasmic reticulum aminopeptidase 2; SNP, single nucleotide polymorphism.

All five SNPs (rs2549782, rs2548538, rs2248374, rs2287988, and rs1056893) were found to be in HWE for all populations studied. Table 1 presents the minor allele frequencies of the *ERAP2* SNPs evaluated in the African-American and Chilean populations studied. Inter-SNP LD testing of the five *ERAP2* SNPs was used to determine the haplotype structure for the Chilean and African-American populations. The African-American population showed two distinct haplotypes based on all five SNPs being in strong LD (D'1.00, R2 > 0.99), GTAGC (0.436) and TAGAT (0.565) (SNP order: rs2549782, rs2548538, rs2248374, rs2287988, rs1056893) (Fig. 1A). The haplotype structure and frequencies identified in African-American fetal and maternal samples is consistent with all previously studied populations (Andrés et al. 2010). The Chilean population did not show distinct haplotypes with the initial analysis of 200 fetal and maternal samples. This initial analysis showed a third haplotype with a low frequency, 1.0%. As 1.0% was at the threshold for exclusion, we expanded the analysis to the full fetal population of 1100 samples. The increased sample size showed two distinct haplotypes, but was based on four SNPs in strong LD (D'1.00, R2 > 0.99), TAGC (0.339) and AGAT (0.661) (SNP order: rs2548538, rs2248374, rs2287988, rs1056893). SNP rs2549782 was not found to be in LD with the other four SNPs in the Chilean population (Fig. 1B). The third haplotype identified in the initial Chilean structure analysis was not present in the expanded analysis, indicating that it was an artifact due to insufficient sample size. All SNPs within each haplotype block, both African-American and Chilean, were found to have strong 2 SNP LD scores, demonstrating that the intermediate markers in the solid spine calculations were in LD with each other. The observation that rs2549782 and rs2248374 are in LD in the African-American population, but not in the Chilean population has important implications for ERAP2 protein expression.

**Table 1:** Minor allele frequencies of the *ERAP2* SNPs in African-American and Chilean population

Population	SNP	Minor allele	Maternal		Neonatal	
	
			Controls	Cases	Controls	Cases
African-American	rs2549782	G	296 (0.43)	176 (0.44)	267 (0.39)	160 (0.41)
	rs2548538	T	41 (0.45)	43 (0.46)	35 (0.38)	46 (0.49)
	rs2248374	A	296 (0.43)	176 (0.44)	533 (0.38)	282 (0.43)
	rs2287988	G	41 (0.45)	43 (0.46)	35 (0.38)	46 (0.49)
	rs1056893	C	38 (0.44)	43 (0.46)	35 (0.38)	46 (0.49)
Chilean	rs2549782	G	399 (0.35)	348 (0.33)	374 (0.33)	351 (0.33)
	rs2548538	T	33 (0.35)	29 (0.31)	393 (0.35)	340 (0.33)
	rs2248374	A	376 (0.33)	348 (0.33)	400 (0.35)	348 (0.33)
	rs2287988	G	33 (0.35)	29 (0.31)	393 (0.35)	339 (0.33)
	rs1056893	C	33 (0.35)	29 (0.31)	389 (0.35)	335 (0.33)

Data are presented as total number (frequency). ERAP2, endoplasmic reticulum aminopeptidase 2; SNPs, single nucleotide polymorphisms.

### Placental ERAP2 and ERAP1 protein expression and rs2248374

**Figure 2:**
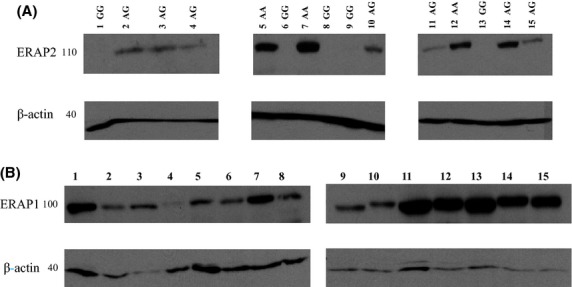
ERAP2 and ERAP1 protein expression with corresponding SNP rs2248374 genotypes in Chilean placental tissue. (A) ERAP2 and β-actin protein expression for 15 Chilean placental basal plate samples (B) ERAP1 and β-actin protein expression for corresponding samples. ERAP2, endoplasmic reticulum aminopeptidase 2; SNP, single nucleotide polymorphism.

In African-Americans, the putative major allele protein 392N should never be expressed as a result of LD with the rs2248374 SNP that results in nonsense-mediated RNA decay (Andrés et al. 2010). This is consistent with the previously reported monoallelic expression of rs2549782 (Bjornsson et al. 2008; Song et al. 2012; Lee et al. 2013). However, because these two SNPs are not in LD in Chilean populations, both the major allele of ERAP2 rs2549782 protein as well as the minor allele protein variant, 392K, should be expressed. To determine if rs2248374 alleles in the Chilean population control ERAP2 protein expression, Western blot analysis on placental extracts from Chilean subjects was performed to determine the relationship between ERAP2 protein expression and *ERAP2* SNPs and/or haplotypes. Western blotting revealed that subjects homozygous for the G allele of rs2248374 (*n* = 5) did not express the 110 kDa ERAP2 protein. Subjects homozygous for the A allele of rs2248374 (*n* = 3) showed greater protein expression than those subjects who were heterozygous with both A and G alleles (*n* = 7) (Fig. 2A). The majority of heterozygous subjects showed expression levels approximately one half of that found in the homozygous A subjects. Thus, rs2248374 has a controlling role in ERAP2 protein expression in Chilean populations as expected from previous reports on other populations. Each rs2248374 genotype was also compared to protein expression of ERAP1, a paralog of ERAP2 that is implicated in functions similar to ERAP2, and no relationship to protein expression was observed (Fig. 2B).

### Genotypic distribution of rs2549782 and rs2248374 alleles in Chilean fetal (neonatal) samples

**Figure 3:**
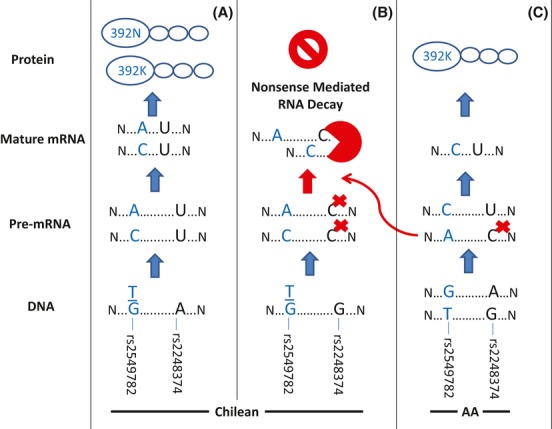
Predicted expression of ERAP2 protein variants based on rs2549782 and rs2248374 SNP alleles in Chilean and African-American populations. (A, B, and C) show the predicted 392 amino acid residues of ERAP2 protein based on the possible DNA sequences on a single chromosome, with respect to the rs2549782 and rs2248374 SNPs, for Chilean and African-American individuals. (A) Chilean individuals with the A allele of rs2248374 are predicted to have both the G and T alleles of rs2549782 on the same chromosome, resulting in the expression of both 392K and 392N, respectively. (B) Chilean individuals with the G allele of rs2248374 are predicted to not express ERAP2 protein due to the G allele coding for a splice site variation that leads to nonsense-mediated RNA decay (C). African-Americans with the A allele of rs2248374 are predicted to always have the G allele of rs2549782 on the same chromosome and, therefore, to only express the 392K ERAP2 protein. African-Americans with the G allele of rs2248374 are predicted to not express ERAP2 protein, and thus not to express the 392N amino acid coded by the T allele of rs2549782, which is always on the same chromosome, due to the G allele of rs2248374 coding for a splice site variation that leads to nonsense-mediated RNA decay. ERAP2, endoplasmic reticulum aminopeptidase 2; SNP, single nucleotide polymorphism.

The haplotype structure in all previously studied populations results in the monoallelic expression of the minor G allele of rs2549782 (Andrés et al. 2010). The lack of LD between these two SNPs in the Chilean population allows for the expression of the major T allele of rs2549782. The rs2549782 SNP encodes an amino acid change (N392K) that alters protein function (Evnouchidou et al. 2012). Figure 3 represents the predicted monoallelic and biallelic expression of the ERAP2 392 amino acid, based on compound genotype of rs2248374 and rs2549782, of the African-American and Chilean populations, respectively. Table 2 presents the Chilean fetal (neonatal) compound genotypes for rs2549782 and rs2248374, the expected expression of total ERAP2 protein based on the rs2248374 splice-site genotype, and the expected 392N expression based on the combination of an A allele of rs2248374 and a T allele of rs2549782. Genotypes did not differ significantly between cases and controls, using a Bonferroni corrected p-value of *P* ≤ 0.05. No fetal (neonatal) sample, neither case nor control, was found to be both homozygous for the rs2248374 AA genotype and homozygous for the rs2549782 TT genotype, which would result in the double expression of the *ERAP2* allele encoding 392N. To determine if the AA,TT genotype was present in the Chilean maternal samples, the full 1100 samples were genotyped for the rs2248374 and 2549782 SNPs. No maternal sample, neither case nor control, was found to be both homozygous for the rs2248374 AA genotype and homozygous for the rs2549782 TT genotype. Furthermore, Table 3 shows observed versus expected counts for rs2248374 AA homozygotes, rs2549782 TT homozygotes, and rs2248374, rs2549782 AA,TT compound homozygotes in Chilean fetal (neonatal), maternal, and total (fetal + maternal) samples. All rs2248374 AA and rs2549782 TT homozygote counts were significantly different from expected. Based on the full population of 2200 samples (fetal + maternal); our study had 65% power to detect the AA,TT compound homozygote at an alpha of 5%.

**Table 2:** *ERAP2* genotype and ERAP2 protein expression

Genotype		Total	Normal	PE	Predicted ERAP2 protein expression	Predicted rs2549782 392N expression

rs2248374	rs2549782					
AA	TT	0	0	0	++	++
AA	GT	90 (0.08)	41 (0.07)	49 (0.09)	++	+
AA	GG	43 (0.09)	28 (0.05)	15 (0.03)	++	−
AG	TT	151 (0.14)	93 (0.16)^1^	58 (0.11)^1^	+	+
AG	GT	248 (0.23)	126 (0.22)	122 (0.23)	+	±
AG	GG	81 (0.07)	41 (0.07)	40 (0.08)	+	−
GG	TT	346 (0.32)	176 (0.31)	170 (0.33)	−	−
GG	GT	138 (0.13)	69 (0.12)	69 (0.13)	−	−
GG	GG	0	0	0	−	−

rs2248374 minor allele A, major allele G; rs2549782 major allele T, minor allele G; population data are presented as total number (frequency within column group). ERAP2 full-length protein expression is predicted by the rs2248374 splice-site SNP, where the A allele is predicted to have full expression and the G allele is predicted to have no expression. ERAP2 protein expression is represented as −, predicted to have no ERAP2 expressed based on two rs2248374 null alleles (GG); +, predicted to have ½ ERAP2 expression compared to full ++ based on heterozygosity of rs2248374 (AG), and ++ predicted to have full expression of ERAP2 protein based on homozygosity for the rs2248374 minor allele (AA). The major T allele of rs2549782 encodes an Asp at position 392 (392N) and the minor G allele encodes a Lys at position 392 (392K). 392N expression is predicted based on the expression of full-length ERAP2 predicted by the rs2248374 genotype and the genotype of rs2549782, which encodes the 392 amino acid protein. 392N expression is represented as – predicted to have no 392N expressed based on homozygosity for the rs2549782 minor allele (GG) or the pairing of the rs2549782 major allele (T) with the rs2248374 null allele (G); ± predicted expression of 392N depends on the phase of the compound genotype of rs2248374 and rs2549782 on each chromosome such that if the A allele of rs2248374 is on the same chromosome as the T allele of rs2549782, 392N is expected to be expressed, or if the G allele of rs2248374 is on the same chromosome as the T allele of rs2549782, 392N is not expected to be expressed; + is predicted to have 1 allele encoding 392N expressed based on the pairing of an A allele of rs2248374 with 1 T allele of rs2549782; and ++ is predicted to have twice the level of 392N expression as compared to + based on both A alleles of rs2248374 being paired with a T allele of rs2549782.

1 AGTT (SNP order rs2248374, rs2549782) is the only compound genotype that was significantly different between cases and controls on initial analysis. However, the significance did not remain with a Bonferroni corrected *P*-value. Therefore, the compound genotype of rs2248374 and rs2549782 did not differ significantly between cases and controls. ERAP2, endoplasmic reticulum aminopeptidase 2; SNP, single nucleotide polymorphism; PE, preeclampsia.

**Table 3:** Observed versus expected genotype counts for *ERAP2* rs2248374 and rs2549782 in Chilean fetuses (neonates), mothers, and the total population (fetuses + mothers)

*ERAP2* genotype		Fetal			Maternal			Total		
			
rs2248374	rs2549782	Observed	Expected	*P*-value	Observed	Expected	*P*-value	Observed	Expected	*P*-value
AA	TT	0	57	5.66 e-14	0	52	8.20 e-13	0	114	<2.2 e-16
AA	TG	90	28	7.79 e-09	81	27	1.69 e-07	130	56	4.50 e-08
AA	GG	43	14	1.71 e-04	44	14	1.14 e-04	59	28	1.16 e-03
AG	TT	151	111	0.01	138	106	0.04	289	221	1.60 e-03
GG	TT	346	215	1.99 e-10	350	214	4.22 e-11	696	429	<2.2 e-16

*P* values presented are for deviations between expected and observed genotypes. ERAP2, endoplasmic reticulum aminopeptidase 2.

### Association of rs2248374 with PE in Chilean mothers and fetuses (neonates)

A case–control design was used to test for associations between the rs2248374 SNP of *ERAP2* and PE status in Chilean maternal and fetal (neonatal) samples. There was no significant association between rs2248374 and risk for PE in either Chilean fetal samples (*P* = 0.46) or maternal samples (*P* = 0.78). Accordingly, Chilean haplotypes were not significantly associated with PE.

### Analysis of epistasis between rs2549782 and rs2248374

The lack of LD between rs2549782 and rs2248374 in Chilean fetal samples allows not only for independent effects of these two SNPs to be measured, but presents the opportunity to assess epistasis between these SNPs. Both rs2549782 and rs2248374 have functional significance, and rs2248374 determines whether rs2549782 of the same chromosome will be expressed. This led us to hypothesize that certain combinations of these two SNPs may be associated with PE in Chilean fetuses (neonates). However, we found that an interaction between rs2549782 and rs2248374 was not associated with PE in Chilean fetal samples (*P* = 0.842).

## Discussion

*ERAP2* has become a gene of interest in PE, and associations have been reported in several populations (Founds et al. 2009; Johnson et al. 2009; Hill et al. 2011a). It is an attractive candidate because it is involved in immune responses, inflammation, and blood pressure control, all of which are dysregulated in PE (Redman and Sargent 2003; Ilekis et al. 2007; Rusterholz et al. 2007). Different SNPs have been associated with PE in different racial groups and between mothers and fetuses, indicating racial and maternal–fetal differences in genetic contribution to PE (Goddard et al. 2007; Parimi et al. 2008; Hill et al. 2011b). We previously reported that the fetal minor G allele of rs2549782 was associated with an increased risk for PE in African-Americans, but not in African-American mothers or Chilean mothers or fetuses (neonates) (Hill et al. 2011a). In-depth analysis by Andrés et al. (2010) demonstrated that two haplotypes (A and B) of *ERAP2*, characterized by five SNPs (rs2549782, rs2548538, rs2248374, rs2287988, and rs1056893) in strong LD, existed in all populations studied. The B haplotype of *ERAP2* was reported to be a null allele, with no expression due to RNA nonsense-mediated decay resulting from a splice-site variant encoded by the rs2248374 SNP (Andrés et al. 2010). The strong LD found in all other populations studied and the effect of rs2248374 on expression of ERAP2 led us to characterize the haplotype structure of our African-American and Chilean samples in order to determine if a difference in structure could have explained the lack of association observed between rs2549782 and PE in the Chilean population.

In contrast to previously studied populations by Andrés et al., and the African-American population we studied, the haplotype structure of the Chilean population only showed strong LD between four of the SNPs (rs2548538, rs2248374, rs2287988, and rs1056893). In the Chilean population, rs2549782 was not part of the haplotype structure that included the splice-site variant encoded by rs2248374. Andrés et al. analyzed diverse populations, including a Toscani population from Italy. The Toscani population maintained the same haplotype structure as the other populations, but it showed increased variation that suggests the potential for divergence (Andrés et al. 2010). Our Chilean population shows a genetic background that is most similar to Spain. It is not unexpected then that the structure of the Chilean population appears to be an extension of the increasing diversity observed in the Toscani population (Andrés et al. 2010), which is a similar Mediterranean European group.

In African-Americans, all five SNPs are in LD so our previously reported association between fetal rs2549782 of *ERAP2* and PE extends such that the fetal GTAGC haplotype of *ERAP2* is associated with increased risk for PE (Hill et al. 2011a). Based on the LD structure, the effects of any one of the SNPs cannot be analyzed independently. As a result, it is unclear whether rs2549782, rs2248374, or both are relevant to PE status. Both rs2549782 and rs2248374 encode functionally significant changes. The rs2549782 SNP encodes an amino acid change (N392K) that alters peptide trimming activity (Evnouchidou et al. 2012) and, as previously stated, rs2248374 encodes a splice-site variant that results in null protein expression (Tanioka et al. 2003; Coulombe-Huntington et al. 2009; Andrés et al. 2010). The lack of LD between rs2549782 and rs2248374 in the Chilean population presented a unique opportunity to study these two SNPs independently, and also raised the possibility that our previous lack of association between *ERAP2* and PE in Chilean mothers and fetuses (neonates) (Hill et al. 2011a) was in fact due to analysis of rs2549782 not extending to the remaining SNPs, notably rs2248374. However, we did not find an association between rs2248374 and PE in either Chilean mothers or fetuses (neonates). Thus, the lack of an association between rs2549782 and PE in Chileans was not simply attributable to rs2549782 not being a surrogate SNP for rs2248374.

The absence of significant LD between the two SNPs also allowed us to determine whether rs2549782 might have an effect on expression of full-length ERAP2 or whether protein expression in placental tissue was indeed regulated by the rs2248374 splice-site variant. Our analysis of Chilean placental tissue confirmed the expression pattern reported by Andrés et al. where the homozygous GG genotype of rs2248374 showed no detectable protein, the AG genotype showed intermediate expression and the AA genotype showed double expression as compared to the heterozygote. ERAP2 expression was not associated with the rs2549782 genotype. It has also been suggested that ERAP1 expression is affected by ERAP2 expression. Our results indicated that ERAP1 expression was not related to either rs2549782 or rs2248374 genotype.

An unanticipated observation of our study was that the homozygous TT genotype of rs2549782 was not found in Chilean fetuses or mothers with the homozygous AA genotype for rs2248374. There are several possible explanations for this observation, one of which is that the compound AA,TT (SNP order rs2248374, rs2549782) is not tolerated. The double homozygous AA,TT genotype is predicted to allow for the expression of both full-length ERAP2 alleles based on the rs2248374 AA genotype and both of those proteins are predicted to have the 392N amino acid based on the rs2549782 TT genotype. In all previously studied populations, the five SNP haplotype structure of *ERAP2* pairs the null G allele of rs2248374 with the major T allele of rs2549782, such that the 392N form of ERAP2 would never be expressed (Andrés et al. 2010). The lack of LD between these two SNPs in the Chilean fetal population allows for the first potential expression of the major T allele.

It is unusual that the ancestral (major) allele of a coding region SNP is not expressed, especially when it is linked to a nonsynonymous amino acid substitution. The amino acid change between the major T and minor G alleles of rs2549782 (N392K) alters antigen processing (Evnouchidou et al. 2012) and, therefore, has significant implications for immune tolerance during pregnancy. The 392N and 392K protein has similar trimming activity for positively charged N-terminal amino acids. However, 392N has about 165-fold greater activity for hydrophobic amino acids compared with the 392K protein. Therefore, this functional change could have a significant impact on antigen presentation in trophoblastic tissue. Increased antigen presentation could make “foreign” proteins of the fetus, those encoded by paternal genotype, more visible to the mother's immune system and activate an increased immune response by the mother against the fetal tissue. A key principle of pregnancy is immune tolerance of the fetus by the mother. These results raise the possibility that increased peptide trimming in trophoblastic tissue resulting from homozygous expression of the allele encoding 392N may not be tolerated.

An alternative explanation for the AA,TT genotype not being detected is the potentially new evolutionary rearrangement of the two *ERAP2* 5-SNP haplotypes (GTAGC and TAGAT; SNP order rs2549782, rs2548538, rs2248374, rs2287988, rs1056893) in Chileans. As suggested by the increasing diversity in the Toscani population, the new 4-SNP haplotype, where rs2549782 is no longer in LD, likely represents an evolutionarily recent rearrangement of the 5-SNP haplotypes. Our results showed that in addition to no Chilean fetuses having the AA,TT compound genotype (SNP order rs2248374, rs2549782), no GG,GG compound genotypes were detected. The AG (SNP order rs2248374, rs2549782) and GT genotypes appear to be the common combinations, whereas the AT and GG combinations may be new in the Chilean population. These combinations would represent the phase, or alleles that reside on the same chromosome. If they do represent relatively new combinations of the two SNPs, they likely have a much lower frequency in the population than the common combinations. Genotyping does not allow for measured phasing and the frequency of the combination (phase) of SNP genotypes is not reflected in single SNP frequencies. Therefore, the actual frequency of the phased combinations (AG vs. GT vs. AT vs. GG) (rs2248374, rs2549782) is unknown. If the new combinations are indeed rare, the Chilean population studied consisting of 2200 individuals might not have been large enough to detect an individual with either the AA,TT or GG,GG compound genotype. This is reflected in the fact that our study only had 65% power to detect the AA,TT compound homozygote in the 2200 individuals tested.

There are several limitations to the current study that will need to be addressed in future work. First, the predicted expression of the 392N form of ERAP2 is based only on rs2549782 and rs2248374 genotypes. It has not been confirmed by mRNA sequence analysis. Second, future studies with a larger sample size are required to establish that the AA,TT (rs2248374, rs2549782) compound genotype is never observed in the Chilean population. Thirdly, our study did not find an association between *ERAP2* and PE in either Chilean mothers or fetuses (neonates). PE is a complex trait, and many genetic variants with small effects may contribute to disease risk. It is possible that *ERAP2* does contribute to PE in Chileans, or that we have not yet assessed the genetic variant associated with risk for PE in this population. Furthermore, genes are predicted to not only exert primary effects, but also to interact with other genes and environmental factors to contribute to disease. It is possible that by not accounting for these interactions in our study, we failed to detect an effect of *ERAP2* in the Chilean population.

In conclusion, we have described a novel haplotype structure for *ERAP2* in a Chilean population. This new structure allowed for the independent analysis of rs2248374 and rs2549782, two SNPs with functional implications for *ERAP2*. We found that, in agreement with previous work (Andrés et al. 2010), full-length ERAP2 expression in Chilean fetal tissue was associated with the rs2248374 genotype. We also found that neither rs2549782 nor rs2248374 was associated with PE in Chilean mothers or fetuses (neonates). Finally, our study failed to observe the AA,TT (rs2248374, rs2549782) in Chilean fetal or maternal samples. This finding raises the possibility that homozygous expression of the 392N form of ERAP2 is not tolerated. Our study disclosed new complexities of the *ERAP2* gene, and further evidence for racial differences in the role of ERAP2 in risk for PE.

## References

[b1] Andrés AM, Dennis MY, Kretzschmar WW, Cannons JL, Lee-Lin SQ, Hurle B (2010). Balancing selection maintains a form of ERAP2 that undergoes nonsense-mediated decay and affects antigen presentation. PLoS Genet.

[b2] Anoymous (2000). Report of the National High Blood Pressure Education Program Working Group on High Blood Pressure in Pregnancy. Am. J. Obstet. Gynecol.

[b3] Anoymous (2002). ACOG practice bulletin. Diagnosis and management of preeclampsia and eclampsia. Number 33, January 2002. Obstet. Gynecol.

[b4] Barrett JC, Fry B, Maller J, Daly MJ (2005). Haploview: analysis and visualization of LD and haplotype maps. Bioinformatics.

[b5] Bjornsson HT, Albert TJ, Ladd-Acosta CM, Green RD, Rongione MA, Middle CM (2008). SNP-specific array-based allele-specific expression analysis. Genome Res.

[b6] Coulombe-Huntington J, Lam KC, Dias C, Majewski J (2009). Fine-scale variation and genetic determinants of alternative splicing across individuals. PLoS Genet.

[b7] Evnouchidou I, Birtley J, Seregin S, Papakyriakou A, Zervoudi E, Samiotaki M (2012). A common single nucleotide polymorphism in endoplasmic reticulum aminopeptidase 2 induces a specificity switch that leads to altered antigen processing. J. Immunol.

[b8] Founds SA, Conley YP, Lyons-Weiler JF, Jeyabalan A, Hogge WA, Conrad KP (2009). Altered global gene expression in first trimester placentas of women destined to develop preeclampsia. Placenta.

[b9] Fruci D, Giacomini P, Nicotra MR, Forloni M, Fraioli R, Saveanu L (2008). Altered expression of endoplasmic reticulum aminopeptidases ERAP1 and ERAP2 in transformed non-lymphoid human tissues. J. Cell. Physiol.

[b10] Goddard KA, Tromp G, Romero R, Olson JM, Lu Q, Xu Z (2007). Candidate-gene association study of mothers with pre-eclampsia, and their infants, analyzing 775 SNPs in 190 genes. Hum. Hered.

[b11] Hill LD, Hilliard DD, York TP, Srinivas S, Kusanovic JP, Gomez R (2011a). Fetal ERAP2 variation is associated with preeclampsia in African Americans in a case-control study. BMC Med. Genet.

[b12] Hill LD, York TP, Kusanovic JP, Gomez R, Eaves LJ, Romero R (2011b). Epistasis between COMT and MTHFR in maternal-fetal dyads increases risk for preeclampsia. PLoS One.

[b13] Ilekis JV, Reddy UM, Roberts JM (2007). Preeclampsia–a pressing problem: an executive summary of a National Institute of Child Health and Human Development workshop. Reprod. Sci.

[b14] Johnson MP, Roten LT, Dyer TD, East CE, Forsmo S, Blangero J (2009). The ERAP2 gene is associated with preeclampsia in Australian and Norwegian populations. Hum. Genet.

[b15] Lee RD, Song MY, Lee JK (2013). Large-scale profiling and identification of potential regulatory mechanisms for allelic gene expression in colorectal cancer cells. Gene.

[b16] Parimi N, Tromp G, Kuivaniemi H, Nien JK, Gomez R, Romero R (2008). Analytical approaches to detect maternal/fetal genotype incompatibilities that increase risk of pre-eclampsia. BMC Med. Genet.

[b17] Purcell S, Neale B, Todd-Brown K, Thomas L, Ferreira MA, Bender D (2007). PLINK: a tool set for whole-genome association and population-based linkage analyses. Am. J. Hum. Genet.

[b18] Redman CW, Sargent IL (2003). “Pre-eclampsia, the placenta and the maternal systemic inflammatory response–a review”. Placenta.

[b19] Rusterholz C, Hahn S, Holzgreve W (2007). Role of placentally produced inflammatory and regulatory cytokines in pregnancy and the etiology of preeclampsia. Semin. Immunopathol.

[b20] Song MY, Kim HE, Kim S, Choi IH, Lee JK (2012). SNP-based large-scale identification of allele-specific gene expression in human B cells. Gene.

[b21] Tanioka T, Hattori A, Masuda S, Nomura Y, Nakayama H, Mizutani S (2003). Human leukocyte-derived arginine aminopeptidase. The third member of the oxytocinase subfamily of aminopeptidases. J. Biol. Chem.

[b22] Tsujimoto M, Hattori A (2005). The oxytocinase subfamily of M1 aminopeptidases. Biochim. Biophys. Acta.

[b23] Wang H, Parry S, Macones G, Sammel MD, Ferrand PE, Kuivaniemi H (2004). Functionally significant SNP MMP8 promoter haplotypes and preterm premature rupture of membranes (PPROM). Hum. Mol. Genet.

[b24] Williams PJ, Broughton Pipkin F (2011). The genetics of pre-eclampsia and other hypertensive disorders of pregnancy. Best Pract. Res. Clin. Obstet. Gynaecol.

[b25] Zhang Y, Cui Y, Zhou Z, Sha J, Li Y, Liu J (2010). Altered global gene expressions of human placentae subjected to assisted reproductive technology treatments. Placenta.

