# A novel *ERAP2* haplotype structure in a Chilean population: implications for ERAP2 protein expression and preeclampsia risk

**DOI:** 10.1002/mgg3.49

**Published:** 2013-11-11

**Authors:** Derek L Vanhille, Lori D Hill, DaShaunda D Hilliard, Eun D Lee, Maria E Teves, Sindhu Srinivas, Juan P Kusanovic, Ricardo Gomez, Efstratios Stratikos, Michal A Elovitz, Roberto Romero, Jerome F Strauss

The original article to which this Corrigendum refers was published in Molecular Genetics and Genomic Medicine 1(2):98–107 (DOI 10.1002/mgg3.13). In this article, references to studies regarding *ERAP2* SNP associations in different ethnic populations were cited incorrectly in the Introduction and Discussion sections. The correct sentences should read:

## Introduction

Certain *ERAP2* single nucleotide polymorphisms (SNPs) have been reported to be associated with an increased risk for PE in different ethnic populations, including rs2549782 (Johnson et al. 2009; Hill et al. 2011a).

## Discussion

*ERAP2* has become a gene of interest in PE, and associations have been reported in several populations (Johnson et al. 2009; Hill et al. 2011a).

The authors regret these errors.

